# The Identification of Differentially Expressed Genes Showing Aberrant Methylation Patterns in Pheochromocytoma by Integrated Bioinformatics Analysis

**DOI:** 10.3389/fgene.2019.01181

**Published:** 2019-11-15

**Authors:** Dengqiang Lin, Jinglai Lin, Xiaoxia Li, Jianping Zhang, Peng Lai, Zhifeng Mao, Li Zhang, Yu Zhu, Yujun Liu

**Affiliations:** ^1^Department of Urology, Xiamen Hospital of Zhongshan Hospital, Fudan University, Xiamen, China; ^2^Department of Radiology, Xiamen Hospital of Zhongshan Hospital, Fudan University, Xiamen, China; ^3^Department of Urology, Ruijin Hospital, Medical School of Shanghai Jiaotong University, Shanghai, China

**Keywords:** pheochromocytoma, bioinformatics, expression, methylation, KCNQ1, SCN2A

## Abstract

Malignant pheochromocytoma (PHEO) can only be fully diagnosed when metastatic foci develop. However, at this point in time, patients gain little benefit from traditional therapeutic methods. Methylation plays an important role in the pathogenesis of PHEO. The aim of this research was to use integrated bioinformatics analysis to identify differentially expressed genes (DEGs) showing aberrant methylation patterns in PHEO and therefore provide further understanding of the molecular mechanisms underlying this condition. Aberrantly methylated DEGs were first identified by using R software (version 3.6) to combine gene expression microarray data (GSE19422) with gene methylation microarray data (GSE43293). An online bioinformatics database (DAVID) was then used to identify all overlapping DEGs showing aberrant methylation; these were annotated and then functional enrichment was ascertained by gene ontology (GO) analysis. The online STRING tool was then used to analyze interactions between all overlapping DEGs showing aberrant methylation; these results were then visualized by Cytoscape (version 3.61). Next, using the cytoHubba plugin within Cytoscape, we identified the top 10 hub genes and found that these were predominantly enriched in pathways related to cancer. Reference to The Cancer Genome Atlas (TCGA) further confirmed our results and further identified an upregulated hypomethylated gene (*SCN2A*) and a downregulated hypermethylated gene (*KCNQ1*). Logistic regression analysis and receiver operating characteristic (ROC) curve analysis indicated that *KCNQ1* and *SCN2A* represent promising differential diagnostic biomarkers between benign and malignant PHEO. Finally, clinical data showed that there were significant differences in the concentrations of potassium and sodium when compared between pre-surgery and post-surgery day 1. These suggest that *KCNQ1* and *SCN2A*, genes that encode potassium and sodium channels, respectively, may serve as putative diagnostic targets for the diagnosis and prognosis of PHEO and therefore facilitate the clinical management of PHEO.

## Introduction

Pheochromocytoma (PHEO) arises from the extra-adrenal sympathetic and parasympathetic ganglia (also referred to as the paraganglioma), as well as the intra-adrenal medulla. This tumor is rare, with a reported incidence of 1 in 300,000 (Else et al., 1993; Lefebvre and Foulkes 2014; Lenders et al., 2014). However, PHEO is a frequent cause of secondary hypertension, a potentially life-threatening cardiovascular complication (Zelinka et al., 2012; Prejbisz et al., 2013). Clinical reports show that up to 36% of patients develop malignancy (Pacak et al., 2007). On the other hand, reports from autopsy research estimate that 0.05–0.1% of cases remain undiagnosed (Jain et al., 2019). Current guidelines for the early treatment of PHEO recommend radical surgical resection. The 5-year survival rate post-surgery in benign cases of PHEO ranges from 84% to 96%, but is less than 50% in malignant cases; the recurrence rate can be as high as 65.45 within 5 years (Schurmeyer et al., 1988; Walther et al., 1999; Kopf et al., 2001; Edstrom Elder et al., 2003). Once PHEO enters an advanced stage, effective treatment modalities are limited, but include radionuclide therapy (^131^I-MIBG) (van Hulsteijn et al., 2014), chemotherapy (a combination of cyclophosphamide, vincristine, and dacarbazine) (Vogel et al., 2014), and external beam radiation therapy (Vogel et al., 2014). However, patients suffering from the advanced stages of PHEO gain little benefit from such treatment modalities. Therefore, there is an urgent need to investigate the key genes involved in the progression of this disease. The identification of new biomarkers could help us to improve the prognosis of patients and facilitate clinical management.

Research studies have identified germline mutations in around one third of patients with PHEO (Lenders and Eisenhofer, 2017) and have identified a range of susceptibility genes, including *RET*, *HIF2A*, *VHL*, *NF1*, *SDHx* (*SDHA*, *SDHB*, *SDHC*, *SDHD*, *SDHAF2*), *FH*, *TMEM127*, and *MAX* (Wallace et al., 1990; Latif et al., 1993; Mulligan et al., 1993; Baysal et al., 2000; Niemann and Muller, 2000; Astuti et al., 2001; Hao et al., 2009; Burnichon et al., 2010; Qin et al., 2010; Comino-Mendez et al., 2011; Castro-Vega et al., 2014). Although genomic variation appears to occur more commonly in PHEO than in any other human tumors (Karagiannis et al., 2007; Fishbein and Nathanson, 2012), research has failed to identify specific genes related to carcinogenesis. Over recent years, the use of microarrays and sequencing has become a promising and effective technique with which to screen hub disease-causing genes and identify biomarkers of diagnostic, prognostic, and therapeutic value. To our knowledge, a complete bioinformatic analysis of PHEO, using the Gene Expression Omnibus (GEO) database and The Cancer Genome Atlas (TCGA), has yet to be carried out, particularly with regards to gene expression and methylation.

In this study, we first identified and screened differentially expressed genes (DEGs) showing aberrant methylation in PHEO by combining gene expression microarray data (GSE19422) and gene methylation microarray data (GSE43293). We then identified 10 core genes showing differential expression and aberrant methylation to act as suitable candidates for further interaction network analysis. TCGA was then used to verify the expression of these core genes and investigate their prognostic value. Our overall goal was to explore new genetic targets that may help us to improve patient outcomes.

## Materials and Methods

### Microarray Data

Two gene expression profiles were downloaded from GEO (www.ncbi.nlm.nih.gov/geo/): platform GPL6480—Agilent-014850 Whole Human Genome Microarray 4x44K G4112F (GSE19422, including 84 PHEO tissues and six normal adrenal tissues); and the gene methylation dataset—Illumina HumanMethylation450 arrays (GSE43293, including 22 PHEO tissues and two normal adrenal tissues).

### Data Processing

All aberrantly methylated DEGs were analyzed with R software (version 3.6) (www.r-project.org/). For DEGS, we used a |log(fold change [FC])| value >1 and an adjusted *P* value <0.05 as cutoff criteria following normalization and background correction with the affyPLM package in R. Data relating to aberrantly methylated genes were first normalized using the beta-mixture quantile dilation (BMIQ) method in the R wateRmelon package. We then used a *β* value >0.2 and an adjusted *P* value <0.05 as cutoff standards.

### Gene Ontology Functional Enrichment Analysis

An online bioinformatics database (DAVID, Database for Annotation, Visualization, and Integrated Discovery, https://david.ncifcrf.gov/) was used to identify all overlapping DEGs showing aberrant methylation. These were annotated and then functional enrichment was ascertained by gene ontology (GO) analysis, including biological processes (BP), molecular function (MF), and cellular component (CC) (Consortium, 2006; Huang da et al., 2009). The GO functional enrichment results were visualized using the ggplot2 package in R.

### Protein–Protein Interaction Network and Module Analysis

The online STRING tool (http://string-db.org) (Park et al., 2009) was used to search for potential correlations among the overlapping DEGs showing aberrant methylation. Cytoscape software (version 3.61; https://cytoscape.org) (Haffner et al., 2017) was then used to build a protein–protein interaction (PPI) network and analyze potential interactions. The cytoHubba plugin and the maximal clique centrality (MCC) method were then used to identify the top 10 hub genes. We then used the MCODE plugin to screen core modules of the PPI network with a standard degree cutoff of 2, a node score cutoff of 0.2, a k-core of 2, and a maximum depth of 100.

### Expression Analysis of Candidate Genes in TCGA

The cBioPortal (www.cbioportal.org/) and UCSC Xena (http://xena.ucsc.edu/welcome-to-ucsc-xena/) platforms, in combination with the TCGA database (TCGA-PCPG), were used to analyze genetic alterations, gene expression levels, and the relationship between expression and methylation. In total, TCGA featured 184 datasets that were available for methylation and expression analysis. We also used the Human Protein Atlas (HPA) database to investigate the expression levels of candidate genes in normal adrenal tissues.

### Kaplan–Meier Survival Analysis for Candidate Genes in TCGA

The Kaplan–Meier plotter (www.kmplot.com/) was used to determine the prognostic value of candidate genes in TCGA. *P* values <0.05 were considered to be statistically significant.

### Clinical Information

With the approval of our institutional ethics review board, we collected clinical information, including tumor size and biochemical data, from 136 patients who underwent adrenalectomy and were subsequently diagnosed with PHEO following surgery. The clinical data (**Supplementary Table 1**) were collected between January 2016 and May 2019 from the Department of Urology in Ruijin Hospital affiliated to the Medical School of Shanghai Jiaotong University in China.

### Statistical Analysis

All data are presented as means ± standard deviation. Statistical analyses were performed with SPSS software (version 23.0;IBM). Bar graphs and scatter diagrams were created by GraphPad Prism 7 software. Data analysis and correlation were carried out using paired *t* tests and either Pearson’s or Spearman’s correlation analysis, as well as line regression analysis. Outliers were analyzed using Spearman’s correlation analysis. We then created a logistic model featuring two selected variables, the expression levels of KCNQ1 and SCN2A, to act as a test for differential diagnosis. Finally, a receiver operating characteristic (ROC) curve was drawn to evaluate the effect of this differential diagnostic test. *P* values <0.05 were considered to be statistically significant.

## Results

### The Identification of Aberrantly Methylated DEGs in PHEO

**Figure 1:**
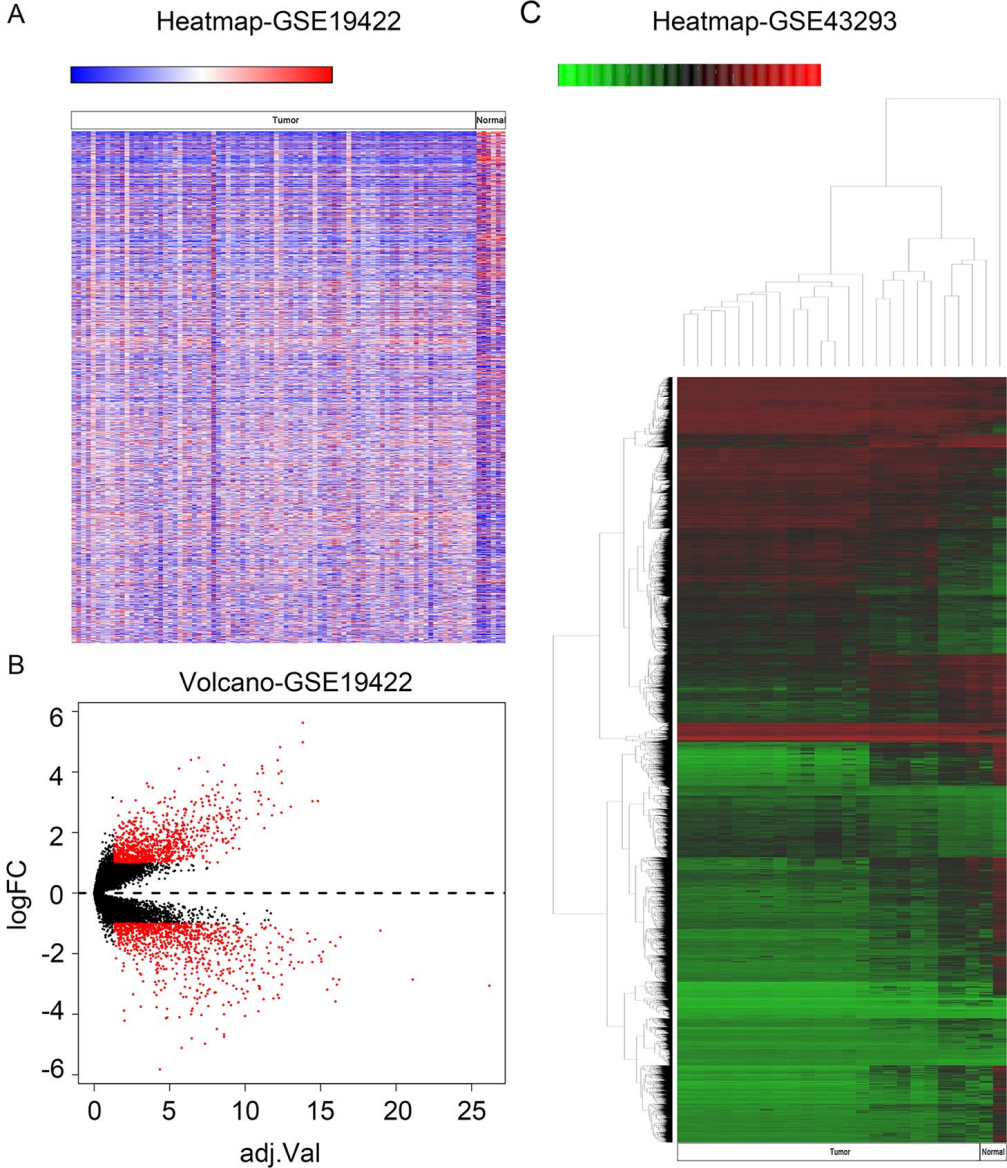
Identification of differentially expressed genes (DEGs) and differentially methylated genes. **(A)** Heat map of DEGs in GSE19422. *Red*, upregulated genes; *blue*, downregulated genes. **(B)** Volcano plot of DEGs in GSE19422 (*red dots*). **(C)** Heat map of DEGs in GSE43293. *Red*, hypermethylated genes; *green*, hypomethylated genes.

**Figure 2:**
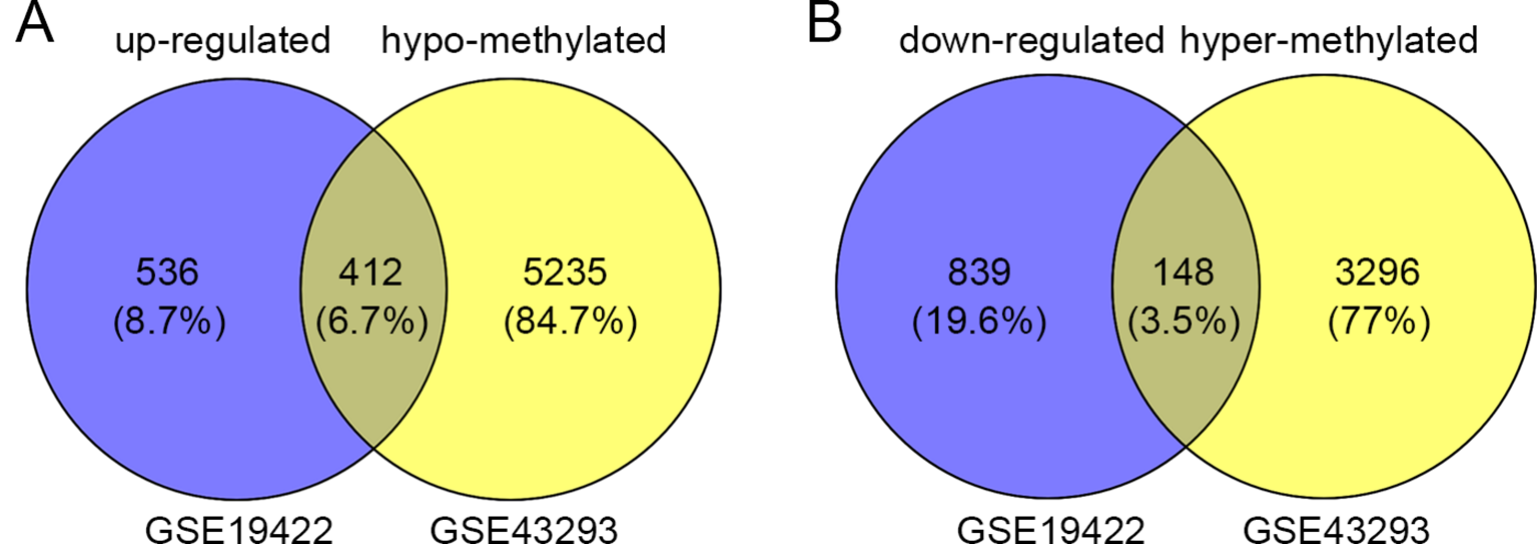
Identification of aberrantly methylated differentially expressed genes (DEGs). **(A)** 412 upregulated and hypomethylated genes were identified. **(B)** 148 downregulated and hypermethylated genes were identified.

In order to identify genes that were differentially expressed in PHEO and normal tissues, we first downloaded the gene expression profile dataset GSE19422 (84 PHEO tissues and six normal tissues) from the NCBI GEO database. Analysis of GSE19422 led to the identification of 1,935 significant DEGs (948 upregulated and 987 downregulated) for further study (**Figures 1A, B**). Methylated data were then standardized in the GSE43293 dataset to further identify 3,444 hypermethylated and 5,660 hypomethylated genes (**Figure 1C**). To identify DEGs showing aberrant methylation, all 948 upregulated genes and 5,660 hypomethylated genes were imported collectively into a Venn diagram. This led to the identification of 412 hypomethylated and highly expressed genes for further analysis (**Figure 2A**). Analysis also identified 148 hypermethylated genes with low expression levels (**Figure 2B**).

### GO Enrichment Analysis of Aberrantly Methylated DEGs by DAVID 6.8

**Figure 3:**
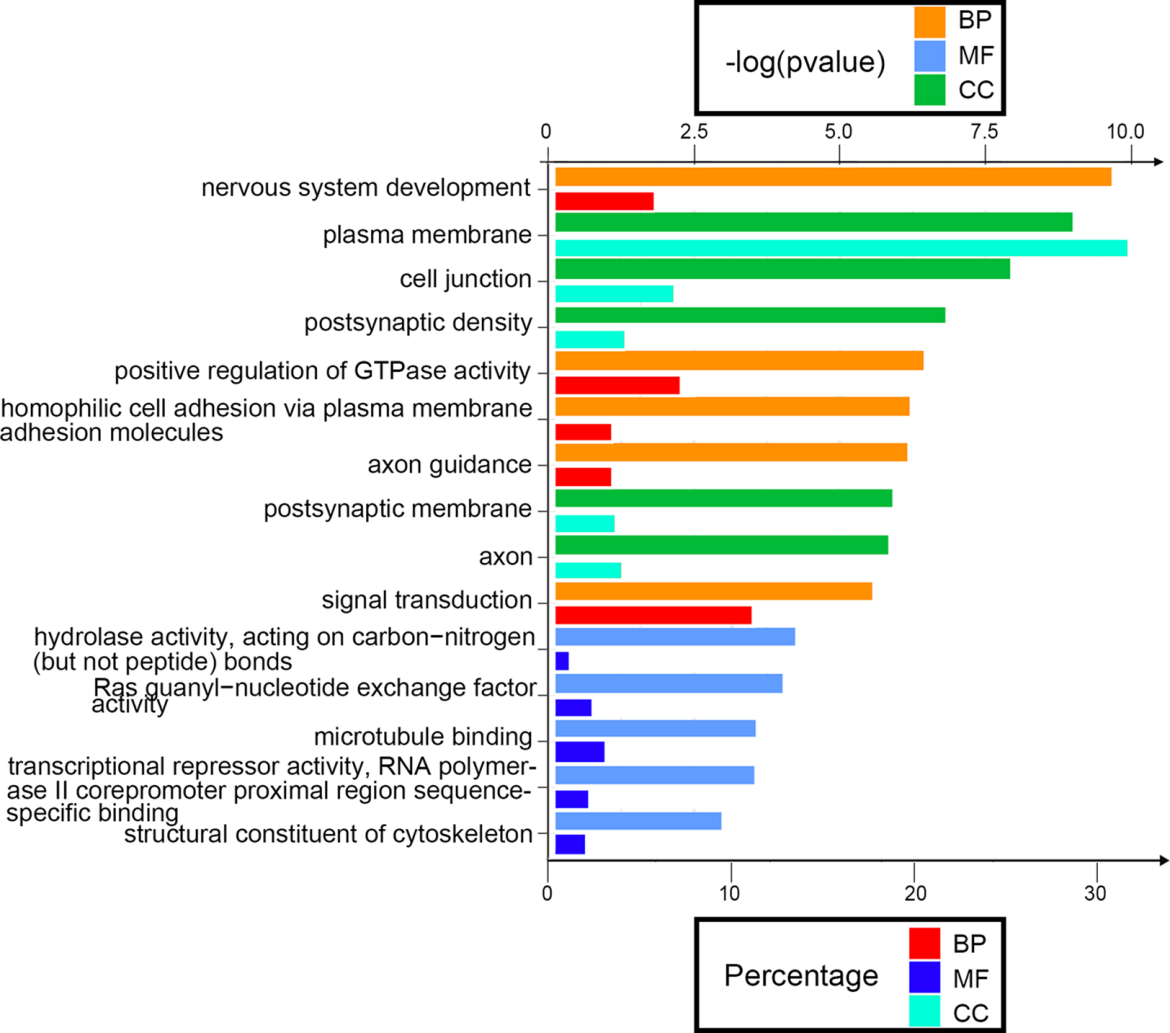
Enrichment analysis of aberrantly methylated differentially expressed genes (DEGs). *BP*, biological processes; *MF*, molecular function; *CC*, cellular component.

Next, we attempted to identify the biological function of the 560 aberrantly methylated DEGs. To do this, we used the DAVID 6.8 online tool to carry out GO functional enrichment analysis. As shown in **Figure 3**, the top 5 functions for BP were as follows: development of the nervous system, the positive regulation of GTPase activity, homophilic cell adhesion *via* plasma membrane adhesion molecules, axonal guidance, and signal transduction. The top 5 functions for MF were as follows: enriched in hydrolase activity, acting on carbon–nitrogen (but not peptide) bonds, Ras guanyl-nucleotide exchange factor activity, microtubule binding, transcriptional repressor activity, RNA polymerase II core promoter proximal region sequence-specific binding, and structural constituent of cytoskeleton. The top 5 locations for CC were plasma membrane, cell junction, postsynaptic membrane, postsynaptic density, and axon.

### The Identification of Hub Genes by Protein–Protein Interaction Analysis Using STRING and Cytoscape 3.61

**Figure 4:**
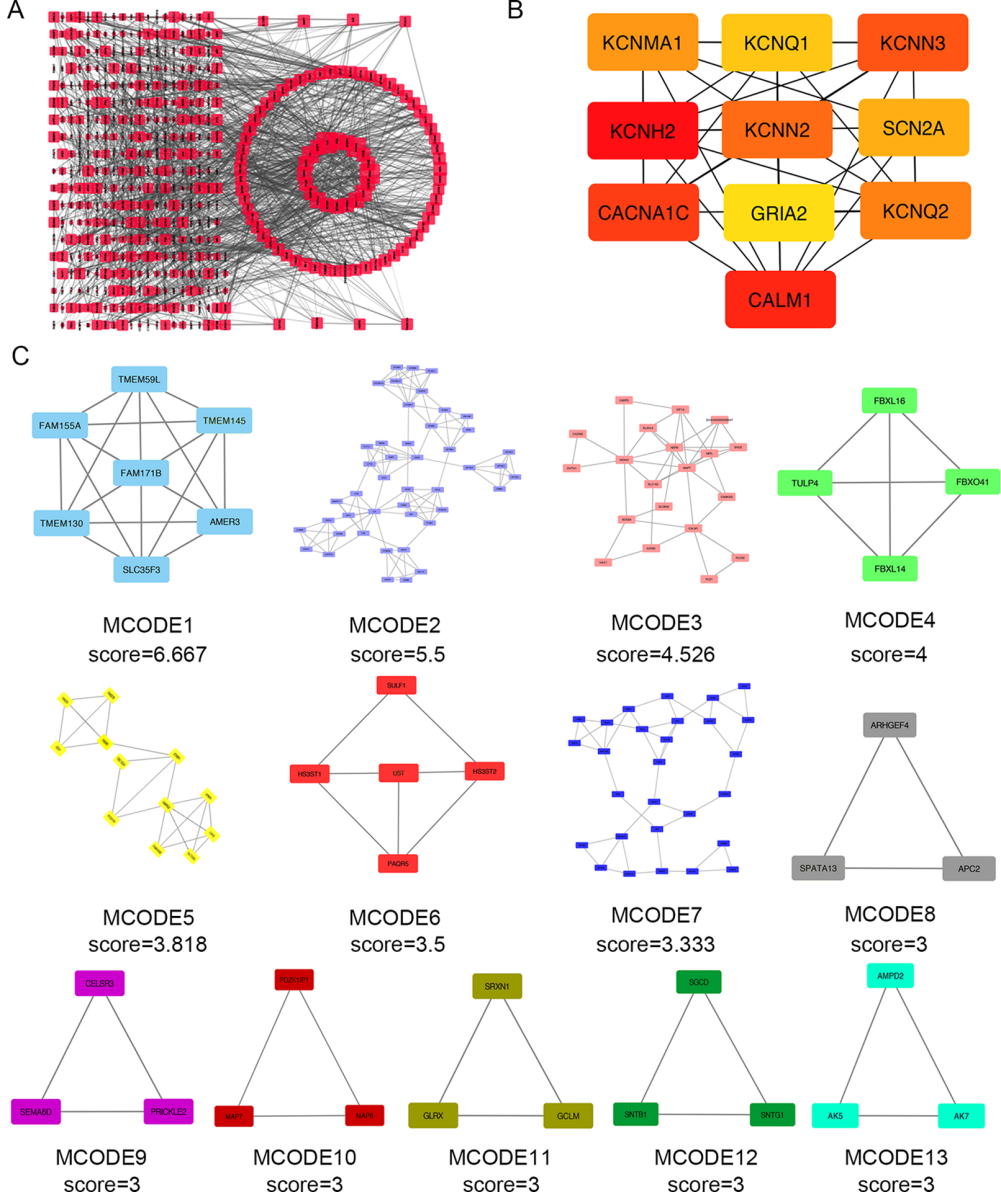
Protein–protein interaction (PPI) network analysis and the identification of hub genes for the aberrantly methylated genes. **(A)** The PPI network included a total of 550 nodes and 1,463 edges. **(B)** The top 10 hub genes were evaluated using the maximal clique centrality method. **(C)** Module analysis for aberrantly methylated DEGs.

**Figure 5:**
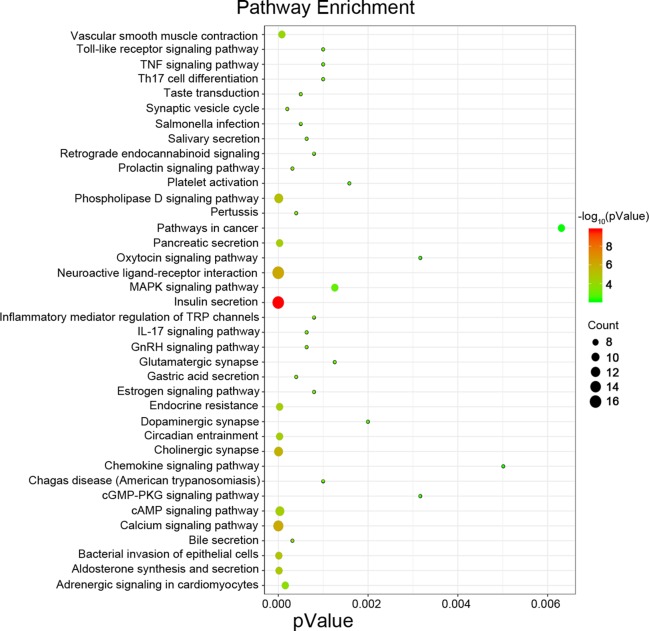
Pathway enrichment analysis for the genes from a core module MCODE 2.

Next, we attempted to identify hub genes among the 560 aberrantly methylated DEGs. To do this, we used PPI analysis and the online STRING platform to examine protein interaction effects among aberrantly methylated DEGs. As illustrated in **Figure 4A**, the PPI network included a total of 550 nodes and 1,463 edges (PPI enrichment *P* < 1.0 × 10^−16^); these results were imported into Cytoscape 3.61 software for visual analysis. Using the cytoHubba plugin and the MCC method, we identified the top 10 hub genes: *CALM1*, *CACNA1C*, *KCNH2*, *KCNQ2*, *KCNMA1*, *KCNN2*, *GRIA2*, *KCNQ1*, *KCNN3*, and *SCN2A* (**Figure 2B**). The MCODE plugin of Cytoscape 3.61 was then used to analyze the whole network; this identified 13 sub-networks (**Figure 2C**). Of these, module 1 achieved the highest score (score: 6.667), while module 2 featured the most hub genes (five in total: *KCNH2*, *KCNMA1*, *KCNN2*, *GRIA2*, *KCNQ1*, and *KCNN3*). Core module analysis indicated that hub genes may play roles in pathways related to cancer, such as the phospholipase D signaling, cAMP signaling, IL-17 signaling, Toll-like receptor signaling, TNF signaling, and MAPK signaling (**Figure 5**). Consequently, these 10 candidate hub genes were selected for further analysis.

### Expression Levels of Candidate Hub Genes in TCGA

**Figure 6:**
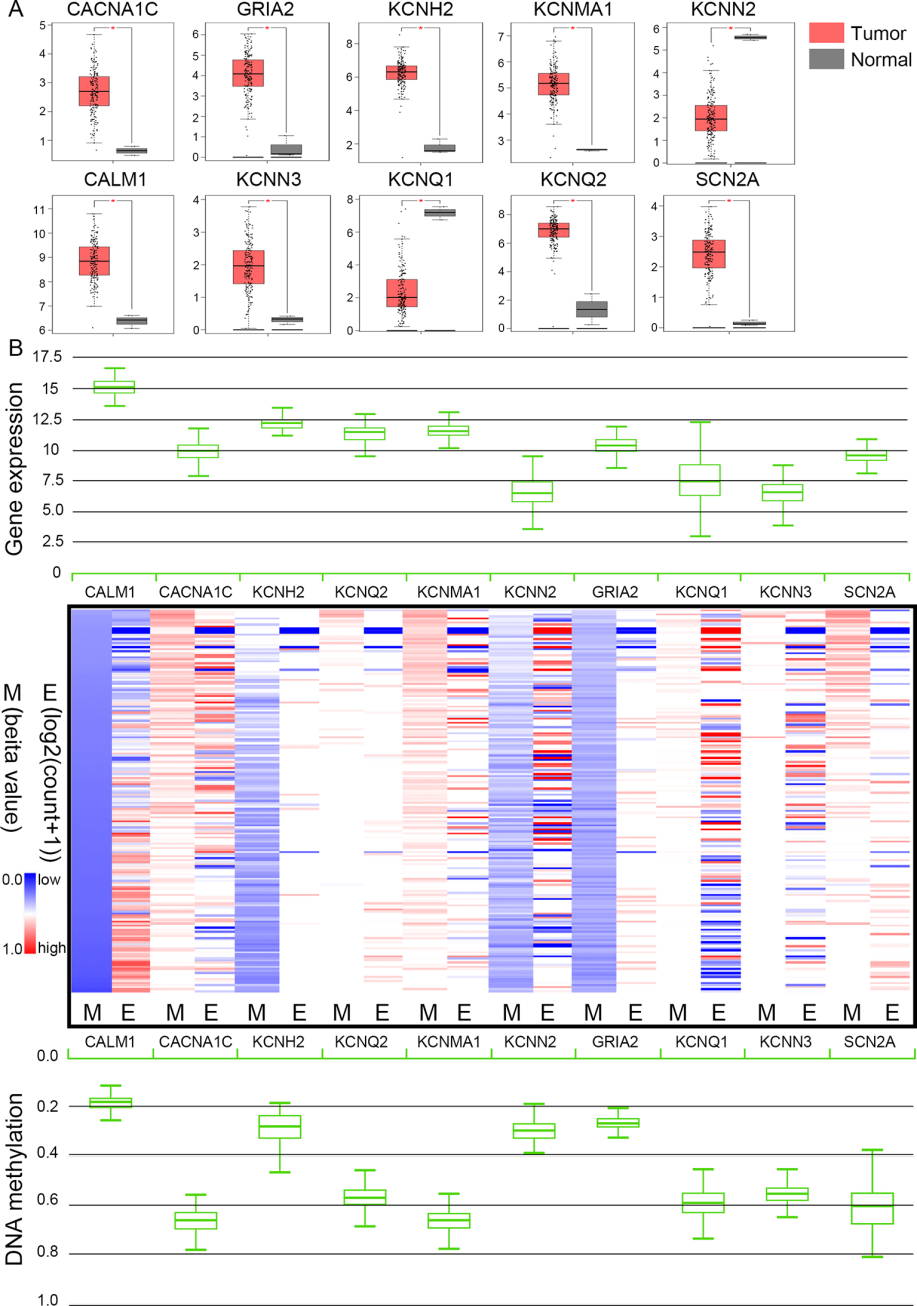
Expression and methylation of 10 hub genes in The Cancer Genome Atlas (TCGA) database. **(A)** Box plots showing 10 hub genes at the mRNA expression level using data from the TCGA database and the GEPIA tool. **(B)** Heat map showing the correlation between the mRNA expression and DNA methylation of the 10 hub genes with the UCSC Xena platform. *M* DNA methylation, *E* mRNA expression. *Red*, upregulated genes in E or hypermethylated genes in M; *blue*, downregulated genes in E or hypomethylated genes in M.

**Figure 7:**
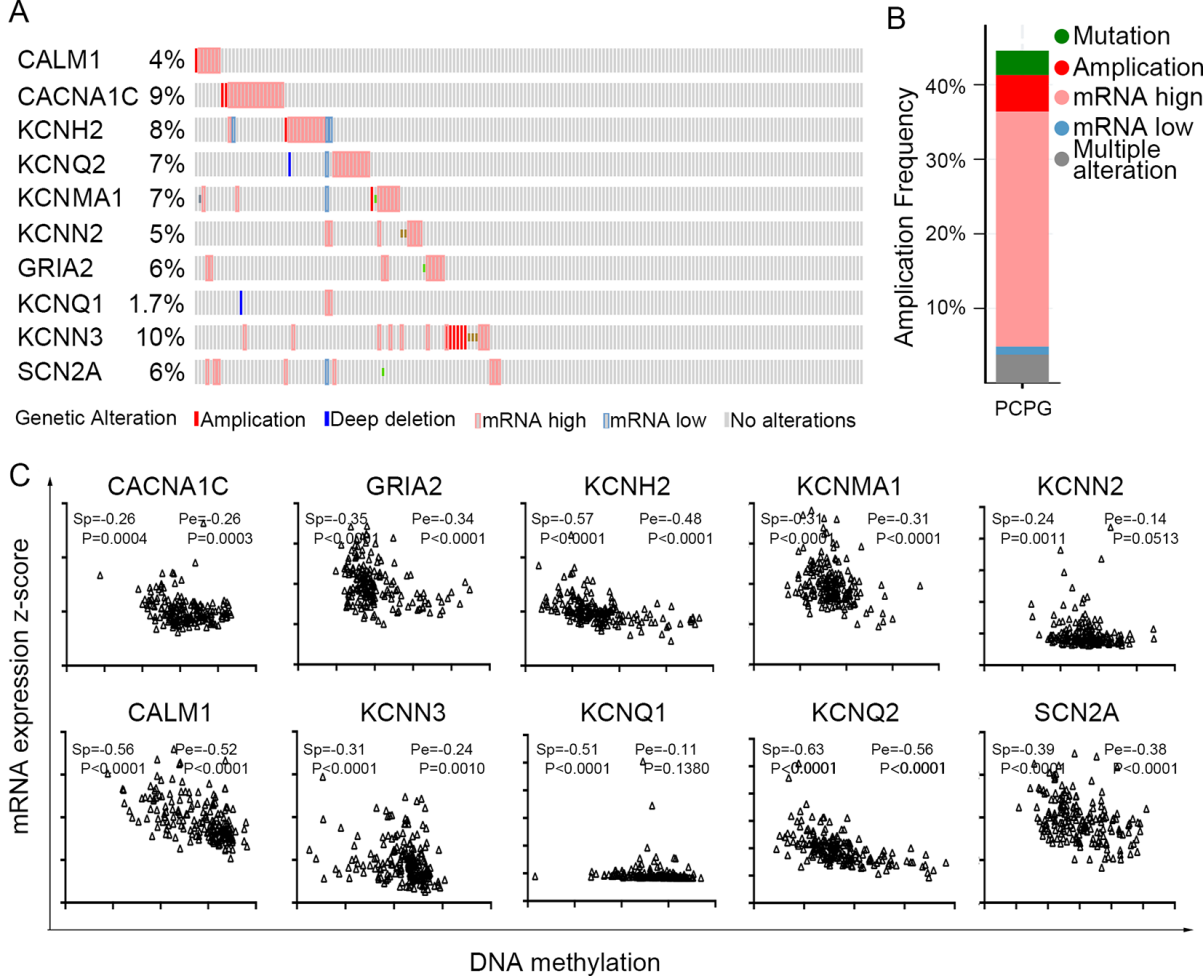
Use of the TCGA database to validate the 10 hub genes. **(A)** Genetic alterations in the 10 hub genes. **(B)** An overview of genetic alteration in the 10 hub genes. **(C)** Correlation between mRNA expression and DNA methylation for the 10 hub genes. *Sp* Spearman correlation analysis, *Pe*: Pearson’s correlation analysis.

The TCGA database was used to further verify our selection of key hub genes. Analysis showed that the 10 hub genes in PHEO tissues showed similar expression levels when compared between the TCGA and the GSE19422 dataset (**Figure 6A**) and similar methylation patterns (**Figure 6B**). As shown in **Figures 7A, B**, these hub genes showed alterations in 44.57% of the 184 cases, including mutation (3.26%) and amplification (4.89%). In addition, we found that the mRNA expression levels of the 10 hub genes showed a significant and negative relationship to the levels of DNA methylation (**Figure 7C**). Collectively, these findings indicated that DNA methylation exerts a significant effect on the progression of PHEO progression by influencing the expression of hub genes.

### The Clinical Value of Candidate Hub Genes in PHEO

**Figure 8:**
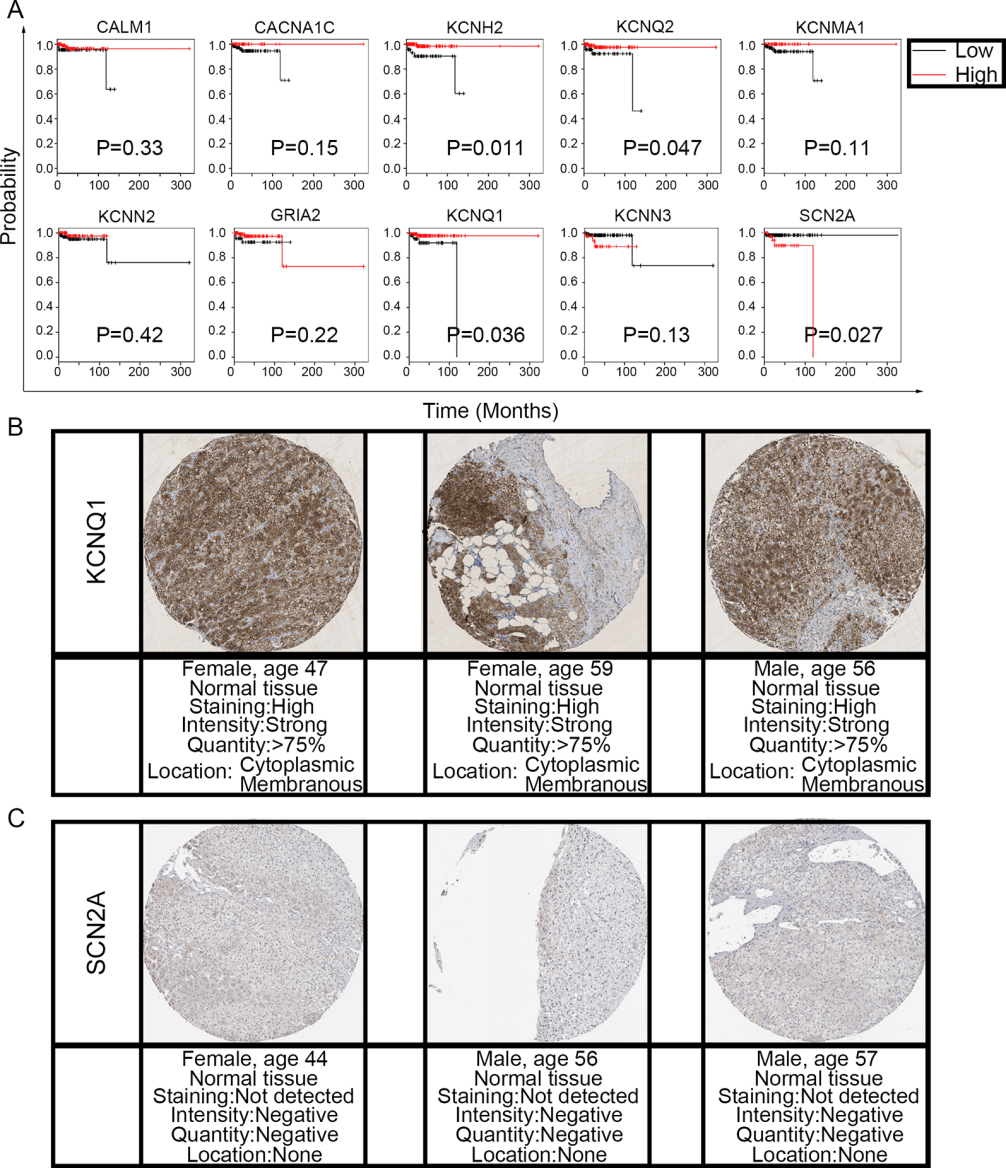
Prognostic value of the 10 hub genes and the expression levels of *KCNQ1* and *SCN2A* in normal tissues. **(A)** Correlation between gene expression and prognosis. **(B)** *KCNQ1* showed strong expression in normal adrenal tissue. **(C)** Normal adrenal tissue was negative for *SCN2A* immunostaining.

**Figure 9:**
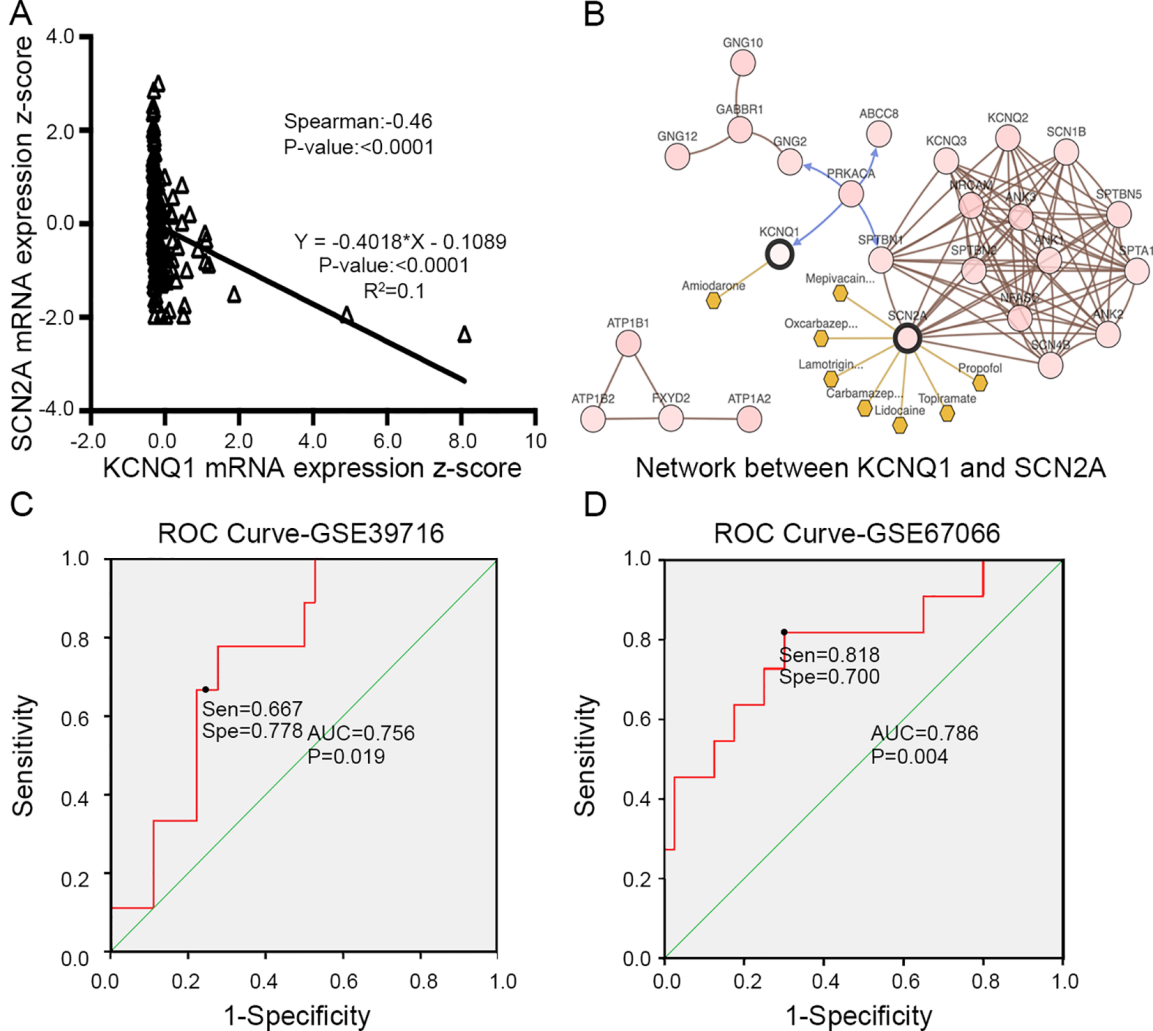
**(A)** Correlation between *KCNQ1* and *SCN2A* expression levels by Spearman’s correlation and line regression analysis. **(B)** Interaction network between *KCNQ1* and *SCN2A*, along with other cancer drugs targeted to *KCNQ1* and *SCN2A*. **(C)** ROC analysis of GSE39716 to discriminate between benign and malignant PHEOs, showing the sensitivity and specificity of this test. **(D)** ROC analysis of GSE67066 to discriminate between benign and malignant PHEOs, showing the sensitivity and specificity of this test. *Sen* sensitivity, *Spe* specificity, *AUC* area under the curve, *ROC* receiver operating characteristic.

To evaluate the prognostic value of the candidate hub genes, we performed survival analysis using the online Kaplan–Meier plotter. **Figure 8A** shows that the overexpression of *KCNH2*, *KCNQ2*, and *KCNQ1* was significantly associated with a good prognosis; in contrast, the overexpression of *SCN2A* was significantly associated with a poor prognosis. Because of the overexpression of *KCNH2* and *KCNQ2* in PHEO when compared with normal tissues, we eliminated these genes in our subsequent analysis (**Figure 8A**). Immunohistochemical staining results from the Human Protein Atlas database indicated that *KCNQ1* showed strong expression levels in normal adrenal tissue (**Figure 8B**); in contrast, *SCN2A* was only expressed in very small levels in normal adrenal tissue (**Figure 8C**). Therefore, we investigated *KCNQ1* and *SCN2A* further to identify their potential therapeutic value. As depicted in **Figure 9A**, the expression levels of *KCNQ1* in PHEO tissues were negatively associated (Spearman’s *r* = −0.46, *P* < 0.0001, and line regression coefficient = −0.4018, *P* < 0.0001) with the expression levels of *SCN2A*, suggesting that patients with PHEO may benefit from interventions targeting one of them. To this end, we established a relationship network (**Figure 9B**), including *KCNQ1* and *SCN2A*, as well as the 50 most frequently altered neighboring genes. Furthermore, some cancer drugs targeted to *KCNQ1* and *SCN2A* were included in the network, some of which are known to exhibit anti-PHEO effects, such as Propofol (Wang et al., 2018) and lidocaine (Tan et al., 2016).

Due to the dilemma posed by the differential diagnosis of benign and malignant PHEOs, we performed logistic regression analysis. We attempted to improve efficiency of differential diagnosis by analyzing two gene expression datasets: GSE39716 (36 benign and nine malignant profiles) and GSE67066 (40 benign and 11 malignant profiles). Two variables, the expression levels of *KCNQ1* and *SCN2A*, were entered into backwards stepwise logistic regression analysis (**Table 1**). *KCNQ1* from the GSE39716 dataset showed the largest relative risk (RR) (50.562, *P* = 0.028), followed by SCN2A from the GSE67066 dataset (4.424, *P* = 0.009).

**Table 1:** Logistic regression analysis.

Dataset	Genes	Level (Ben vs. Mal)	*B*	*P* value	RR (95%CI)
GSE39716	*KCNQ1*	7.12 ± 0.46 vs. 6.82 ± 0.22	3.923	0.028	50.562 (1.542–1658.4)
	*SCN2A*	8.18 ± 0.82 vs. 8.33 ± 0.48	0.434	0.556	1.544 (0.364–6.554)
GSE67066	*KCNQ1*	7.39 ± 0.83 vs. 7.26 ± 0.68	0.95	0.097	2.586 (0.841–7.957)
	*SCN2A*	7.49 ± 0.84 vs. 6.50 ± 1.13	1.487	0.009	4.424 (1.444–13.556)

Ben, benign PHEO; Mal, malignant PHEO; B, regression coefficient; RR, relative risk; CI, confidence interval.

Then, we created a ROC curve to evaluate the value of this procedure for differential diagnosis. The area under the ROC curves for GSE39716 (**Figure 9C**) and GSE67066 (**Figure 9D**) were 0.756 [*P* = 0.019, 95% confidence interval (CI) = 0.606–0.906] and 0.786 (*P* = 0.004, 95% CI = 0.619–0.954), respectively. Corresponding sensitivity and specificity were 0.667 and 0.778, and 0.818 and 0.7, respectively, indicating that *KCNQ1* and *SCN2A* may represent promising differential diagnostic biomarkers.

**Figure 10:**
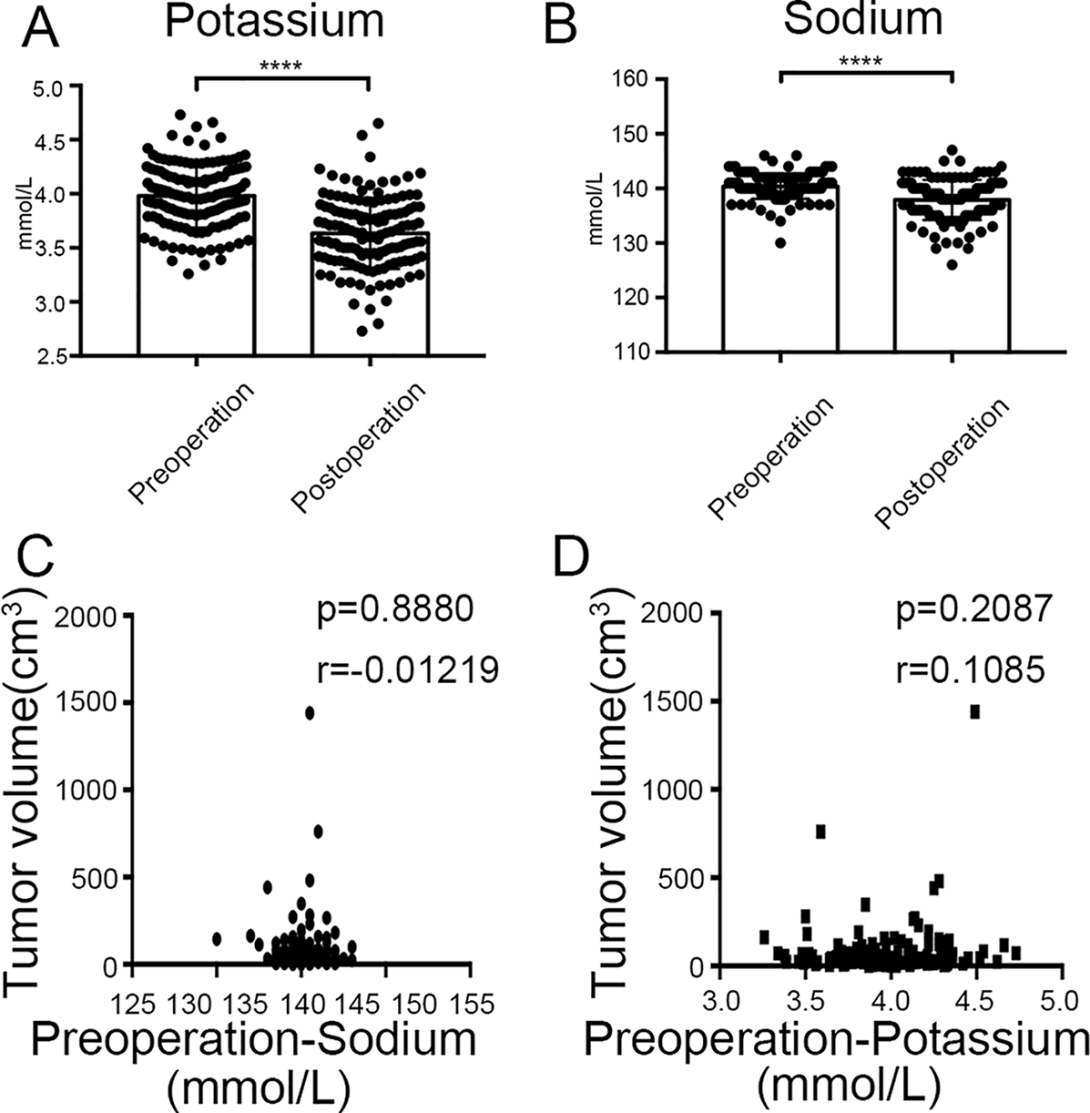
Clinical values of *KCNQ1* and *SCN2A*. **(A)** Histogram showing a significant difference between the pre-surgical status and post-surgery day 1 for potassium concentration. ****P < 0.0001. **(B)** Histogram showing a significant difference between the pre-surgical status and post-surgery day 1 for sodium concentration. ****P < 0.0001. **(C)** Correlation between tumor volume and pre-surgical sodium concentration. **(D)** Correlation between tumor volume and pre-surgical potassium concentration.

Finally, we found that there was significant difference between potassium concentration (3.98 ± 0.29 mmol/l vs. 3.63 ± 0.33 mmol/l, *P* < 0.0001) and sodium concentration (140.36 ± 2.26 mmol/l vs. 137.90 ± 3.66 mmol/l, *P* < 0.0001) when compared between pre-surgery and post-surgery day 1 (**Figures 10A, B**). However, the concentrations of potassium and sodium prior to surgery were not associated with tumor size (**Figures 10C, D**).

## Discussion

Despite significant effort, there is still little we can do to improve the prognosis of patients with PHEO, particularly in malignant cases. Consequently, there is a clear need to explore the specific pathogenesis of this disease and identify core genes or proteins that may facilitate clinical diagnosis and treatment. As a free and commonly used resource, the NCBI GEO database features a significant body of microarray proﬁling and next-generation sequencing for a variety of human tumors. Using this database, we downloaded gene expression microarray data (GSE19422) and gene methylation microarray data (GSE43293) for further analysis. In particular, we screened two of the most important hub genes, a downregulated hypermethylated gene (*KCNQ1*) and an upregulated hypomethylated gene (*SCN2A*) in PHEO tissues, both of which were further validated by the TCGA database. Functional enrichment results indicated that these hub genes played a role in the pathogenesis and progression of PHEO through certain pathways. We aimed to provide a new perspective for the pathogenesis, diagnosis, and treatment of PHEO, thus leading to improved patient outcomes.

Using R software, we identified a total of 560 aberrantly methylated DEGs. GO enrichment analysis further indicated that aberrantly methylated DEGs were predominantly involved in cancer-related biological processes, such as the positive regulation of GTPase activity, homophilic cell adhesion *via* plasma membrane adhesion molecules, axonal guidance, and signal transduction. By cycling between an inactive GDP-bound and an active GTP-bound state, the family of GTPases can act as molecular switches and are involved in a range of cellular processes, including cell proliferation, apoptosis, and migration (Olson et al., 1995; Vega and Ridley, 2008; Cherfils and Zeghouf, 2013; Croise et al., 2014; Mack and Georgiou, 2014). The relative effects of GTPases depend on whether they exert action during the initiation or progression of tumors (Ellenbroek and Collard, 2007; Orgaz et al., 2014). The most commonly investigated members of the family of GTPases are RhoA, Cdc42, and Rac1. In a previous study, Croise et al. (2016) reported that PHEO was associated with the reduced activity of Cdc42 and Rac1, and the reduced expression of two Rho-GEFs, FARP1 and ARHGEF1. Our own previous research demonstrated that it inhibited PHEO progression that promotes adhesion molecules, E-cadherin and β-catenin, translocation from cytoplasm to membrane (Lin et al., 2019).

Visualization of the PPI network using Cytoscape software identified a total of 550 nodes and 1,463 edges, thus indicating that almost all of the aberrantly methylated DEGs interacted with each other, either directly or indirectly. These data imply that by manipulating the expression of core genes, it may be possible to interfere with the initiation and progression of PHEO. To this end, we used the cytoHubba plugin to identify the top 10 key genes: *CALM1*, *CACNA1C*, *KCNH2*, *KCNQ2*, *KCNMA1*, *KCNN2*, *GRIA2*, *KCNQ1*, *KCNN3*, and *SCN2A*. Similar to the GEO database, there were similar patterns of expression and methylation for these 10 core genes in PHEO when compared with normal tissues in the TCGA database, such as the downregulated and hypermethylated genes *KCNN2* and *KCNQ1*. In total, 44.57% of the 184 PHEO tissues showed genetic alterations in *KCNN2* and *KCNQ1*. These results demonstrated that these 10 core genes may play important roles in the initiation and progression of PHEO. However, only two core genes, *KCNQ1* and *SCN2A*, showed any potential prognostic value when we considered their expression patterns in PHEO.

The *KCNQ1* gene is located on chromosome 11 and has 16 exons and 15 introns. This gene encodes for the pore-forming α-subunit of a voltage-gated potassium channel that allows potassium to ﬂow out of the cell membrane following depolarization. Under physiological conditions, this process maintains homeostasis with regards to ion concentration, cell volume, and pH (Felipe et al., 2006; Huang and Jan, 2014). An increasing body of evidence now supports the essential role of potassium channels in the initiation and progression of tumors, particularly in colorectal cancer (den Uil et al., 2016; Rapetti-Mauss et al., 2017), hepatocellular carcinoma (Fan et al., 2018), and gastric cancer (Liu et al., 2015). Research carried out by Rapetti-Mauss et al. indicated that *KCNQ1* is a target gene for the Wnt/β-catenin pathway and that the loss of *KCNQ1* promoted the disruption of cell–cell contact, thus contributing to EMT (epithelial–mesenchymal transition), cell proliferation, and invasion in colorectal cancer (Rapetti-Mauss et al., 2017). In a previous study, we demonstrated that ApoG2, a small molecular inhibitor, could inhibit PHEO cell migration and invasion by promoting the translocation of E-cadherin and β-catenin from the cytoplasm to the membrane dependent and that this process depended on downregulation of the PI3K/AKT pathway. This suggested that the regulation of β-catenin by *KCNQ1* may play a similar role in the metastasis of PHEO (Lin et al., 2019). Although the rate of *KCNQ1* mutation was only 1.7% (3/179), the expression level of *KCNQ1* was closely associated with the prognosis of patients with PHEO. Based on our current findings, we speculate that the methylation rate of *KCNQ1* might be more relevant than the rate of DNA mutation; this requires verification by further research.

In addition, we hypothesize that *KCNQ1*, as a potassium channel gene, could also influence the levels of potassium. As expected, analysis of our clinical data showed a significant difference for potassium concentration when compared between the pre-surgical state and post-surgery day 1. However, it remains unknown as to whether the concentration of potassium could serve as a prognostic biomarker or not. This is because the levels of potassium can be influenced by a range of factors, including, but not limited to, the progression of PHEO. Furthermore, it is not clear whether cutoff points for potassium concentration would be instructive in clinical practice. These points need to be addressed in future research.

Voltage-gated sodium channels are transmembrane glycoprotein complexes composed of a large α-subunit with 24 transmembrane domains and one or more regulatory β-subunits. The *SCN2A* gene is located on chromosome 2 and has 31 exons that encode one member of the sodium channel α-subunit gene family. Several previous publications have reported an association between *SCN2A* gene mutation and a variety of seizure types (Liu et al., 2015). However, mutation of the gene has not been associated with pathogenesis of tumors, including PHEO. However, analysis of our clinical data revealed a significant difference for sodium concentration when compared between the pre-surgical state and post-surgery day 1, thus suggesting that the pre-surgical concentration was influenced by the tumor. Furthermore, the rate of mutation in the *SCN2A* gene was as high as 6% (10/179) and its expression levels were closely associated with the prognosis of patients with PHEO. Consequently, the biological role and clinical value of *SCN2A* in PHEO clearly warrant further investigation.

## Conclusion

In summary, we used two microarray datasets (GSE19422 and GSE43293) to identify a number of important DEGs showing aberrant methylation in PHEO-related pathways. These findings may help us to develop a better understanding of how genetic alterations are involved in the initiation and progression of PHEO and identify which genes and pathways we should investigate further. Most importantly, we showed that two of the DEGs showing aberrant methylation (*KCNQ1* and *SCN2A*) represent potential biomarkers for the prognosis of patients with PHEO and may help in differential diagnosis between benign and malignant tissues. Consequently, *KCNQ1* and *SCN2A* represent valuable targets for the diagnosis and treatment of PHEO.

## Data Availability Statement

Publicly available datasets were analyzed in this study. This data can be found here: GSE19422, GSE43293, GSE39716, GSE67066.

## Author Contributions

DL, JL, and XL contributed equally to this work and should be considered as co-ﬁrst authors. DL conceived and designed the study. DL and JL analyzed the data. DL and XL prepared the figures and wrote the text for the main manuscript. JZ and PL provided technical guidance. ZM and LZ revised the manuscript. YZ and YL provided funding support. All authors reviewed the manuscript and approved the final version for publication.

## Funding

This study was supported by the National Natural Science Foundation of China (reference number 81272936) and the Shanghai Nature Science Foundation (no. 17ZR1417300).

## Conflict of Interest

The authors declare that the research was conducted in the absence of any commercial or financial relationships that could be construed as a potential conflict of interest.

## Supplementary Material

Supplementary Table 1 Biochemical level of patients.
